# A nomogram to predict the risk of hepatic encephalopathy after transjugular intrahepatic portosystemic shunt in Cirrhotic Patients

**DOI:** 10.1038/s41598-020-65227-2

**Published:** 2020-06-10

**Authors:** Xiaochun Yin, Feng Zhang, Huiwen Guo, Chunyan Peng, Wei Zhang, Jiangqiang Xiao, Yi Wang, Xiaoping Zou, Ming Zhang, Yuzheng Zhuge

**Affiliations:** 1 Department of Gastroenterology, Nanjing Drum Tower Hospital Clinical College of Nanjing Medical University, Nanjing, Jiangsu China; 2 Department of Gastroenterology, Nanjing Drum Tower Hospital, The Affiliated Hospital of Nanjing University Medical School, Nanjing, Jiangsu China

**Keywords:** Gastrointestinal bleeding, Portal hypertension

## Abstract

Background and Aim: Hepatic encephalopathy (HE) is a serious complication of decompensated liver cirrhosis, affecting the prognosis of patients underwent transjugular intrahepatic portosystemic shunts (TIPS). We aim to create a nomogram to predict hepatic encephalopathy- free survivals (HEFS) after TIPS in cirrhotic patients and select appropriate candidates for TIPS. Methods: Cirrhotic patients underwent TIPS from 2015 to 2018 in our department were included. Multivariable Cox regression was conducted to estimate the predictors of overt HE (OHE) after TIPS within one year. A nomogram based on the Cox proportional hazard model using data from a retrospective training cohort (70% of the patients) was developed. Then the prediction model was validated in the remaining 30% patients by Harrell’s C-indexes, ROC curves and calibration plots. Results: Of 373 patients, 117 developed postoperative OHE (31.4%). The training and validation groups comprised 83 (31.4%) and 34 (31.2%) patients, respectively. The cumulative survival rates of patients with HE at 1, 2 and 3 years were 90%, 83% and 76%, respectively. The nomogram included the following variables: age, Child-Turcotte-Pugh class (CTP class), diabetes mellitus (DM), serum creatinine and serum sodium (C-index = 0.772). The C-index for HEFS prediction was 0.773 for the validation cohort. The ROC for predicting HEFS was 0.809 and 0.783, respectively. Conclusions: We created a nomogram of predicting postoperative HEFS in cirrhotic patients received TIPS. This nomogram could be an important tool of HE risk prediction before TIPS to guide the therapeutic strategy in cirrhotic patients.

## Introduction

Transjugular intrahepatic portosystemic shunts (TIPS), has been widely used to treat portal hypertension related complications, including refractory ascites and esophageal and gastric variceal bleeding. However, postoperative complications, in particular hepatic encephalopathy (HE) after TIPS, remain a major setback, which seriously affects the prognosis of cirrhotic patients and significantly increases the risk of mortality^1,2^. Previous study suggests that the incidence of HE after TIPS using covered stents was not increased compared with that of bare stents, but the 1-year postoperative incidence of HE is still up to 35%^3^.

Hepatic encephalopathy (HE) is one of serious complications in the progression of liver disease, increasing rates of readmission and mortality. Ammonia intoxication hypothesis is one of the principal pathogenesis of hepatic encephalopathy^4^. According to the EASL/AASLD guidelines, HE can be classified as covert hepatic encephalopathy (CHE) and overt hepatic encephalopathy (OHE)^5^. HE occurs as a complication, affecting up to 20% of decompensated cirrhotic patients. The patients submitted to TIPS are particularly prompt to develop HE, increasing to 10–50%^6^.

Previous studies have reported that several factors including older age, diabetes mellitus, higher MELD score, previous HE^7^, the stent diameter and proton pump inhibitors treatment^8^ were related to the high risk of developing HE. A meta-analysis^9^ suggested that advanced age, Child-Pugh score and history of HE were predictors of post-TIPS HE in patients with portal hypertension (especially non-alcoholic cirrhosis). In particular, Yousif *et al*.^10^ suggested that lower 25(OH) D level increased the incidence of HE and SBP in cirrhotic patients. But it remains difficult to identify patients at risk. Therefore, it is necessary to build a prediction model that can forecast risk of post-TIPS HE in patients with cirrhosis.

The nomogram is recognized as a reliable tool for constructing an intuitive statistical predictive model to quantify the risk of a clinical event^11^. Each variable included in the nomogram is assigned a value based on its prognostic significance. The aim of our study was to create a prediction nomogram model of OHE in cirrhotic patients treated by TIPS due to esophageal and gastric variceal bleeding (EGVB).

## Results

### Patients characteristics

**Figure 1:**
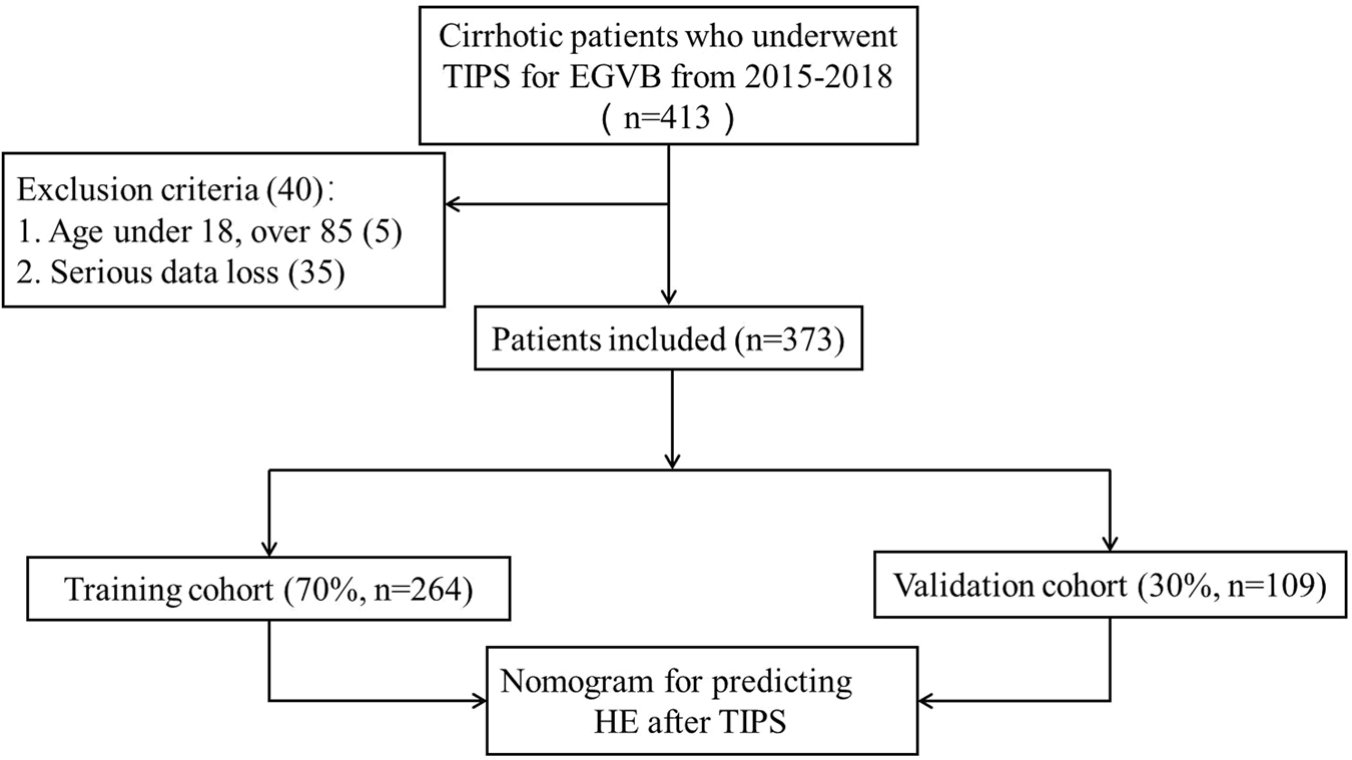
Flow chart. Abbreviation: OHE: Overt Hepatic Encephalopathy; TIPS: Trasjugular Intrahepatic Portosystemic Shunt; EGVB: esophageal and gastric variceal bleeding.

Among 413 cirrhotic patients treated by TIPS for EGVB from January 2015 to December 2018 at our center, forty of the patients were excluded for serious data loss (35) and age under 18 or over 85 (5). Among 373 patients enrolled, 264 patients (70%) were included in the training cohort, and 109 patients (30%) were included in the validation cohort, as shown in Fig. 1. Descriptive characteristics for the overall population are listed in Table 1 and Supplement Table 2. The median follow-up was 37 months (1-60 months). Of all 373 patients, a total of 117 (31.4%) had post-TIPS HE during the follow-up period, and 103 (27.6%) had HE within 12 months. During one year of follow-up, 31.4% (83/264) of the patients in the development cohort and 31.2% (34/109) in the validation cohort had developed overt hepatic encephalopathy (OHE) after TIPS. At the end of follow-up, a total of 68 (18.2%) patients died. The cumulative survival rates of patients with HE at 1, 2 and 3 years were 90%, 83% and 76%, respectively. From Supplement Table 1, we can find that gender, pro-TIPS HE, etiology of liver cirrhosis, total bilirubin and technical data of TIPS procedure were comparable between HE and Non-HE group (P > 0.05). Diabetic patients developed more HE comparing to non-diabetic patients (32.5% VS 16.0%; P  = 0.002). In terms of liver function damage, the mean baseline Child-Turcotte-Pugh (CTP) score of HE group was higher compared to that of Non- HE group. The incidence of OHE also increased with increasing age and serum creatinine. Comparing with Non-HE group, serum sodium level in HE group was significantly lower (p < 0.05).

**Table 1:** General characteristics of training (n = 264) and Validation cohorts (n = 109).

Patient’s characteristics	Training Cohort (n = 264)	Validation Cohort (n = 109)
Gender: male	161(61.0)	68(62.4)
Etiology: Viral/Alcoholic/Autoimmune/Others	161/19/29/55	63/8/15/23
Ascites: No/light/medium/heavy	57/101/70/36	25/33/42/9
Spleen: Yes	208(78.8)	91(83.5)
DM	56(21.2)	21(19.3)
Pre-TIPS OHE	8(3)	4(3.7)
Age	56.88 ± 12.04	55.39 ± 10.45
**Pre-TIPS**		
CTP score	7.21 ± 1.27	7.46 ± 1.37
CTP class A/B/C	68/183/13	24/77/8
MELD-Na	11.92 ± 19.28	14.50 ± 29.49
ALT(U/L)	54.90 ± 297.66	33.29 ± 54.39
AST(U/L)	50.82 ± 172.01	37.23 ± 28.41
TB(ummol/L)	20.99 ± 19.40	23.33 ± 15.69
Scr(ummol/L)	63.56 ± 19.95	70.24 ± 25.78
ALB(g/L)	32.62 ± 4.33	32.78 ± 4.51
Serum sodium(ummol/L)	129.94 ± 4.14	139.87 ± 4.05
PT(s)	14.86 ± 1.86	14.56 ± 2.10
INR	1.29 ± 0.16	1.27 ± 0.28
Fib(g/L)	1.60 ± 0.67	1.55 ± 0.58
WBC(*10^9/L)	3.55 ± 2.50	3.67 ± 2.73
PLT(*10^9/L)	94.65 ± 75.04	84.63 ± 79.44

Ascites: Light: patients generally have abdominal distension, the ascites can only be detected by an ultrasound examination, and the depth is less than 3 cm. Medium: the patient often has moderate and symmetrical abdominal distension, and the depth is 3–10 cm. Heavy: the patient has significant bloating. The ascites detected by ultrasound occupies the entire abdominal cavity, and the depth is more than 10 cm. Data are expressed as mean, number (%), or mean (standard deviation).

Abbreviation: MELD: Model of End-Stage Liver Disease; CTP: Child-Turcotte-Pugh; DM: diabetes mellitus; HE: Hepatic Encepholapathy; PT: Prothrombin time; TB: Total Bilirubin; ALB: Albumin; WBC: white blood cells; AST: aspartate transaminase; ALT: alanine transaminase; INR: international normalized ratio; PLT: platelet; Scr: serum creatinine.

### Identification of prognostic factors for 1-yr post-TIPS HE

Further, we confirm independent prognostic factors for post-TIPS HE by using multivariate regression methods. Hepatic encephalopathy occurred in 26.5% of patients within one year in training cohort. Table 2 displays the univariate and multivariable Cox regression including clinical and laboratory data. We used Cox regression analysis to test 1 year hepatic encephaloapthy-free survival (HEFS) rates after TIPS implantation. Finally, multivariate analysis indicated that age, together with DM, CTP class, serum creatinine and cerum sodium were independent risk factors for hepatic encephalopathy(P < 0.05). Table 3 shows the factors contributing to the final model to predict the risk of developing OHE. The cumulative survival rate of remaining free of HE was 73.4% within 1 year.

**Table 2:** Univariate and Multivariate cox regression analysis of 1-yr HE in patients underwent TIPS (training cohort, N = 264).

Variable	Univariate Analysis		Multivariate Analysis	
	HR (95% CI)	p Value	HR (95% CI)	p-Value
DM	2.572 (1.588–4.163)	<0.001	1.844 (1.059-3.211)	0.030
Age	1.035 (1.014–1.057)	0.001	1.027 (1.002-1.052)	0.033
CTP score	1.307 (1.088–1.569)	0.004	0.833 (0.550-1.260)	0.386
CTP class		0.004		0.042
A				
B	2.157 (1.097–4.240)	0.026	3.240 (0.964–5.893)	0.057
C	5.021(1.908–13.217)	0.001	6.678 (1.679–8.889)	0.012
MELD score	1.010 (0.902–1.130)	0.865		
MELD-Na score	0.994 (0.972–1.016)	0.580		
Serum sodium (ummol/L)	0.911(0.872-0.951)	<0.001	0.935 (0.883-0.989)	0.019
Serum Creatinine (ummol/L)	1.019 (1.009–1.029)	<0.001	1.013 (1.002-1.025)	0.021
PT (s)	0.996 (0.877–1.131)	0.950		
TBil (umol/L)	1.007 (1.000–1.015)	0.059		
Albumin (g/L)	1.000 (0.948–1.056)	0.993		
WBC(*10^9/L)	1.004 (0.917–1.098)	0.939		
PLT(*10^9/L)	1.001 (0.998–1.004)	0.516		
Pre-TIPS NH3	1.003 (0.991–1.016)	0.586		
Ascites		0.006		0.338
No				
Light	0.663 (0.293-1.496)	0.322	1.114 (0.345-3.599)	0.856
Medium	1.883 (1.059–3.347)	0.031	1.173 (0.552-2.495)	0.678
Heavy	2.248 (1.158-4.364)	0.017	1.920 (0.884-4.169)	0.099
SPV	0.975 (0.944-1.007)	0.128		
PV	1.019 (0.992-1.047)	0.168		

Abbreviations: DM: Diabetes Mellitus; WBC: white blood cells; PLT: platelet; PT: Prothrombin time; ALT: alanine transaminase; AST: aspartate transaminase; CTP: Child Turcotte Pugh score; MELD: Model for End-Stage Liver Disease; SPV: Splenic Vein Velocity; PV: Portal Velocity; CI: confidence interval; HR: hazard ratio.

**Table 3:** Factors contributing to the final model.

Variables	HR (95% CI)	p Value
Age	1.029 (1.010-1.049)	0.023
CTP class		0.004
A		
B	2.478 (1.154-5.321)	0.020
C	6.414 (2.109-8.143)	0.001
Serum sodium (ummol/L)	0.938 (0.888-0.990)	0.020
Serum Creatinine (ummol/L)	1.014 (1.003-1.026)	0.012
DM	1.860 (1.076-3.214)	0.026

*P* < 0.05 means statistically significant; CTP score: Child Turcotte Pugh score; DM: Diabetes Mellitus.

### Derivation of the prognostic nomogram

**Figure 2:**
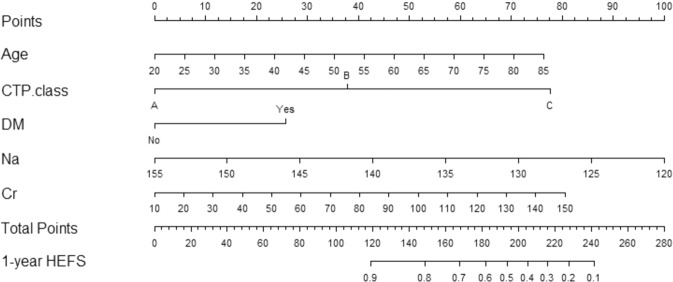
Nomogram for predicting cirrhotic patients with OHE who underwent TIPS based on baseline prognostic factors. Abbreviations: DM: diabetes mellitus; CTP. calss: Child Turcotte Pugh score; Scr: Serum Creatinine; Na: serum sodium; HEFS: Hepatic Encephalopathy- Free Survivals.

**Figure 3:**
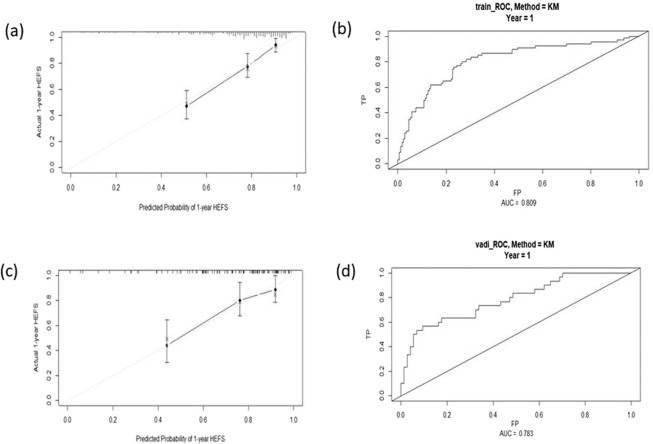
Calibration curves and ROC curves in the primary data and after applying this nomogram in the validation: (**a**) Train cohort (N = 264)-Calibration curve; (**b**) Train cohort (N = 264) - ROC curve; (**c**) Validation cohort (N = 109)-Calibration curve; (**d**) Validation cohort (N = 109)-ROC curve. Abbreviations: HEFS: Hepatic Encephalopathy- Free Survivals.

Nomogram-derived probabilities of 1-yr hepatic encephalopathy- free survivals (HEFS) after TIPS were plotted. The c-index of nomograms for prediction of HEFS were 0.772 and 0.773 in the training and validation cohort. The nomogram and calibration curves are plotted as shown in Figs. 2 and 3. This model contains five variables, which located on each variable axis. We can get the point received for the value of each variable by draw a line up to point axis, and the sum of these points corresponds to the total point axis. Finally, we can determine the likelihood for each individual of 1-yr HEFS by drawing a line downward to the survival axes.

### Validation of the prognostic nomogram

Five prognostic factors were retained in the final model (Fig. 2). The C-index and AUC for the training cohort is 0.772 and 0.809, respectively, which prove that the accuracy and reliability of this nomogram model are relatively high. Calibration and ROC curves were plotted to evaluate the prediction effect of the nomogram (Fig. 3). The prognostic ability of the model was acceptable with a C-index of 0.773 and AUC of 0.783 in validation cohort. The calibration curve also shows that there is only a limited deviation from the ideal prediction model (Fig. 3a,c).

## Discussion

The present study is the first time to establish a nomogram to predict the risk of hepatic encephalopathy (HE) after TIPS procedure. Risk factors contributing to the model included age, DM, CTP class, serum sodium and serum creatinine. HE has attracted increasing attention as one of the common complications after TIPS, which is an important risk factor influencing the prognosis of cirrhotic patients and increasing the rate of death^12^. In our study, a total of 68 (18.2%) patients died. The cumulative survival rates of patients with HE at 1, 2 and 3 years were 90%, 83% and 76%, respectively. For early HE after TIPS, recent studies have shown that recurrence of OHE is associated with an increased risk of mortality in patients with cirrhosis who undergo TIPS^13^. However, some researchers believe that there is no significant correlation between HE and the long-term prognosis of patients^14^. At present, the relationship between HE after TIPS and the long-term survival of patients is still controversial. In our study, we found that the long-term survival of patients with HE after TIPS surgery was poorer than that of patients without HE (3-year survival: 76% vs 90%). Some symptoms of HE are irreversible and have an impact on the long-term quality of life. For high-risk patients, awareness of HE should be raised, and treatment measures should be actively adopted. In our study we saw that 31.4% of patients developed hepatic encephalopathy after TIPS placement, which is agreement with previous observations (10–50%)^6^. Cumulative risk of hepatic encephalopathy within one year after TIPS is about 26.6%. Given that only overt hepatic encephalopathy (OHE) was considered in our study, the whole incidence of HE is lower than that in other studies^15^. Minimal hepatic encephalopathy (MHE) is highly prevalent. Previous studies^8,16^ have reported that about 40% of hospitalized cirrhotic patients have MHE, which may be higher in the disease progression stage.

In our study, the independent predictors for HE incidence were age, CTP class, diabetes mellitus, serum sodium and creatinine after multivariate analyses. Patients with lower serum sodium and higher Child-Pugh score before TIPS might be more probably attacked by early post-TIPS HE which indicated worse long-term survival. Previous studies demonstrated that hyponatremia could affect brain metabolism and increase the risk of hepatic encephalopathy^17–20^. Hyponatremia could worsen the neurological symptoms, which is correlated with the change of osmolality pressure and sodium concentration in the extracellular fluid. The International Society for Hepatic Encephalopathy and Nitrogen Metabolism (ISHEN)^21^ points out that hyponatremia should be prevented as far as possible, avoiding exacerbating the progression of HE. Serum creatinine was also independently associated with HE in cirrhotic patients after TIPS. Renal function including serum creatinine and urea nitrogen involved in in ammonia metabolism, is related to cognitive impairment in cirrhosis^22^.

In addition to CTP class, MELD or MELD-Na score are now widely used to evaluate liver function for the end-stage liver disease and predict mortality of liver failure before or after operation. Serum sodium and creatinine are important parts of MELD-Na score. In this study, serum sodium and creatinine had a strong effect on the OHE rate. HE is an important feature of liver failure, which prompts poor prognosis. This is consistent with our research that patients with higher CTP score (hazard ratio: 1.307, 95% C*I*: 1.088–1.569; *P* = 0.004) are particularly prompt to develop HE. With the increase of child-pugh class, the survival rate of patients without hepatic encephalopathy in 1 year decreased significantly.

In addition, DM was connected with developing post-TIPS OHE, which was confirmed in various studies^23–25^. In our study, we only talked about type 2 diabetes mellitus (T2DM), “which was diagnosed based” on the 1999 World Health Organization criteria^26^. Systemic inflammatory and high protein catabolism and ammonia production may be a potential mechanism. Increased glutaminase activity of liver, kidney and small intestine is also involved in hyperammonemia. In addition, constipation is more common in elderly diabetic patients owing to a prolonged duodenum cecal transit time. Age is also proven to be associated with an increase of HE-related mortality. Aging brain appears to be more responsive to the toxic metabolite including plasma ammonia. And besides, with the increase of age, intestinal empting is prolonged, and constipation and intestinal flora imbalance are more likely occur. All of these are associated with the onset of HE.

On one hand our study identified these risk factors associated with hepatic encephalopathy after TIPS placement. Furthermore, these risk factors are further quantified and presented in the form of nomogram, which is beneficial to clinical practice. Nomogram is a simple and advanced statistical method. One of the advantages of nomograms is that they can be used to assess individual risks based on patient characteristics and disease characteristics. Nomograms have previously been used to predict survival outcomes for patients with various cancer-related diseases^27–29^. The development of tools that predict the prognosis of each patient may be helpful in directing individualized therapy. Malinchoc et al. established a nomogram for predicting 3-month mortality after TIPS in 2000^30^. In this paper, the nomogram scoring system for predicting hepatic encephalopathy- free survival within one year after TIPS has a good survival prediction effect(C-index = 0.772, ROC = 0.809), providing an alternative visual risk prediction chart for the clinic.

The nomograms validated in this study may not be suitable for all patients, but the establishment of a clinical prediction model based on the risk of HE after TIPS would rather serve to heighten awareness of the possibility of post-TIPS HE and make timely adjustments to individualized treatment schemes. According to the risk factors of HE shown in the nomogram model, more targeted prevention and treatment strategies should be implemented, for example, enhancing the awareness of early HE screening for elderly and diabetic patients and strengthening blood sugar management. In the clinical treatment process, comprehensive treatment methods should be adopted to improve various indexes in CTP scoring standards, reduce CTP scoring and grading before TIPS, and thus reduce the incidence of HE after TIPS. For patients with higher risks of HE occurrence, active prevention and treatment after TIPS, such as lactulose and rifaximin therapy, may achieve better quality of life and long-term survival^31^.

However, some limitations remain to be concerned in the present study. First, our retrospective study is from single center, so more prospective multicenter studies in the future may help to further improve the prediction ability of the model. It will be better to validate the model with data from another institution. Second, in this study, we concerned only on overt hepatic encephalopathy and data on mild encephalopathy or covert hepatic encephalopathy are lacking. In spite of these drawbacks, this study firstly builds a nomogram of predicting post-TIPS HE in cirrhotic patients.

In conclusion, our study develops a prediction nomogram of 1-yr HEFS in cirrhotic patients who underwent TIPS for EGVB. Much work remains to do to refine the nomogram and to evaluate any other factors related to the developments of hepatic encephalopathy.

## Methods

### Participants

Patients diagnosed with cirrhosis and successfully underwent TIPS implantation for EGVB from January 2015 to December 2018 were enrolled in the study. Patients under the age of 18 or above 80, with any neurological disease and severe data loss were excluded. Cirrhosis was diagnosed by a combination of clinic features, laboratory findings, imaging evidence and endoscopy and conformed by liver biopsy as necessary. They were randomized into two groups using R Studio version. About 70% (264 of 373 patients) of the patients were selected as the model group to establish a prediction model, and the remaining 30% (109 of 373 patients) were used as the validation group to test the model (Fig. 1).

The study was performed in accordance with the Declaration of Helsinki and was approved by the Ethics Committee of the Nanjing Drum Tower Hospital, Nanjing, Jiangsu, China. All patients signed written informed consent for every procedure.

### Data collection

Preoperative data were collected, including relevant examinations results within 7 days prior to TIPS. Complete blood count, kidney function test, serum electrolyte, plasma ammonia level, liver function test, prothrombin time, international normalized ratio, abdominal ultrasonic Doppler and previous history were recorded in all patients. Additionally, the history of preoperative hepatic encephalopathy were reviewed and verified by more than two experienced liver specialists according to West‐Haven criteria.

### TIPS procedure

TIPS implantation technique has been described previously^32^. In brief, the procedure is performed via the jugular vein. A catheter was placed through the jugular vein to the inferior vena cava or hepatic vein. A balloon (Fluency; Bard) was used to dilate the intrahepatic parenchymal tract. During procedure, covered stents (Fluency; Bard, New Jersey, USA) or bare stents ((Luminexx; Bard, New Jersey, USA) or a composite stent of covered and bare metal stent was inserted in the channel, which were dilated to full nominal diameter. Portal vein pressure was measured before and after the stent placement. Besides, tissue glue and some coils (Cook, Bloomington, Indiana, USA) were used to embolize the prominent gastric varices observed during TIPS. Direct portography was then performed to assess the patency of portal venous system.

### Evaluation of hepatic encephalopathy

All patients were followed up at 1, 3, 6 and 12months after TIPS, and then subsequent follow-up performed once a year through observation of clinical symptoms. Investigations including blood ammonia levels, transaminase and ultrasound were performed as necessary. The mental state of the patients was assessed at each follow-up. Hepatic encephalopathy was diagnosed according to the West‐Haven criteria^5^ and the feature of overt HE episodes (>=grade 2 according to West-Haven scoring) were record. The patients and their families were trained to contact the doctors if any mental symptoms appeared, including mental and behavior changes. Besides, hepatology nurses in our department keep in regular contact with the patients and their families after TIPS insertion.

### Statistical analysis

Statistical analysis was performed using SPSS 22.0 (IBM, USA). For continuous variables, t-test was used. Categorical variables were compared with the *F* test. A multivariate regression analysis was conducted to determine statistically risk factors for hepatic encephalopathy, calculating the relative ratios and 95% CI of independent prognostic factors. The significance level was established as 0.05. Nomogram was performed using the “rms” and “survival” packages in R 3.2.5 software (http://www.r-project.org). Calibration curves with average K–M estimates were then plotted to evaluate the performance of nomogram. Bootstrap method was used for model validation. We performed receiver‐operating characteristic (ROC) curve using “pROC” package to explore the predictive efficiency of nomogram model.

## Supplementary information

Supplementary information.
