# Supplementary material for: A nomogram to predict the risk of hepatic encephalopathy after transjugular intrahepatic portosystemic shunt in Cirrhotic Patients
Source: Sci Rep. 2020 Jun 10;10:9381. doi: 10.1038/s41598-020-65227-2 (PMC7287049; doi:10.1038/s41598-020-65227-2)
Supplement: Supplementary file 1 — Supplementary information. [file 41598_2020_65227_MOESM1_ESM.pdf]

A nomogram to predict the risk of hepatic encephalopathy after transjugular intrahepatic portosystemic shunt in Cirrhotic Patients

Xiaochun Yin[1a], Feng Zhang[1a], Huiwen Guo[1], Chunyan Peng[2], Wei Zhang[2], Jiangqiang Xiao[2], Yi Wang[2], Xiaoping Zou[2], Ming Zhang[2]*, Yuzheng Zhuge[1]*

[1] Department of Gastroenterology, Nanjing Drum Tower Hospital Clinical College of Nanjing Medical University, Nanjing, Jiangsu, China; 2423874375@qq.com (X.Y.); fzdndx@126.com (F.Z.); 1420389818@qq.com (H.G.)

[2] Department of Gastroenterology, Nanjing Drum Tower Hospital, the Affiliated Hospital of Nanjing University Medical School, Nanjing, Jiangsu, China; springpcy@aliyun.com (C.P); kimmysai@126.com (W.Z.); josedy@126.com (J.X.); wangyired@foxmail.com (Y.W.); 13770771661@163.com (X.Z.)

* Correspondence: 13851743262@163.com (M.Z.); yuzheng9111963@aliyun.com (Y.Z.); Tel.: +86-025-8310-5206 or +86-159-9628-9206 (Y.Z.); Fax: +86-025-8330-4616 (Y.Z.)

a The authors contributed equally to this work.

Supplementary Table 1 The Characteristics of training (n=264) and validation cohort (n=109)

Patient's characteristics	Training Cohort (n=264)	Validation Cohort (n=109)
**TIPS**		
**Size: 6/7/8/10(mm)**	69/41/153/1	19/19/70/1
**Pre-PVP(mmHg)**	29.67±5.77	29.52±6.17
**Post-PVP(mmHg)**	20.19±5.47	21.40±5.49
**PVP drop(mmHg)**	7.92±6.35	7.19±8.15
**Post-TIPS**		
**CTP score**	7.43±2.00	7.71±1.71
**ALT(U/L)**	65.22±152.89	60.24±87.22
**AST(U/L)**	54.94±85.44	54.39±48.63
**TB(umol/L)**	35.15±31.72	44.08±61.18
**Scr(umol/L)**	56.93±23.17	61.40±28.64
**ALB(g/L)**	29.85±9.11	29.92±7.15
**PT(s)**	16.15±2.33	16.65±5.27
**INR**	1.51±1.45	1.45±0.44
**WBC(*10^9/L)**	5.06±3.59	5.38±4.62
**PLT(*10^9/L)**	76.97±52.12	71.50±73.67
**NH3(mmol/L)**	34.61±22.77	35.79±21.37
**HE**	83(31.4%)	34(31.2%)

Data are expressed as mean, number (%), or mean (standard deviation). CTP: Child-Turcotte-Pugh; HE:

Hepatic Encepholapathy; PT: Prothrombin time; TB: Total Bilirubin; ALB: Albumin; WBC: white blood cells; AST: aspartate transaminase; ALT: alanine transaminase; INR: international normalized ratio; PLT: platelet; Scr: serum creatinine; PVP: Portal Vein Preassure.

Supplementary Table 2 Baseline demographic and clinical characteristics of the training cohort (N=264)

	**Hepatic encephalopathy**		
	Yes(N=83)	No(N=181)	P
**Gender: male**	52(62.7)	109(60.2)	0.707
**Age**	60.01 ±10.83	55.44 ±12.32	0.004
**DM**	27(32.5)	29(16.0)	0.002
**Pro-TIPS OHE**	3(3.6)	5(2.8)	0.712
**Etiology: Viral/ Alcoholic/ Autoimmune/ Others**	46/7/9/21	115/11/20/35	0.648
**Ascites: No/light/medium/heavy**	11/26/28/18	46/75/42/18	0.004
**CTP score**	7.49 ±1.23	7.08 ±1.27	0.015
**CTP class: A/B/C**	13/62/8	55/121/5	0.005
**MELD score**	9.98 ±1.93	9.98 ±2.21	0.978
**MELD-Na**	10.51 ±3.17	10.06 ±23.12	0.432
**ALT(U/L)**	24.38 ±21.86	68.90 ±358.63	0.260

**AST(U/L)**	32.71±23.69	59.12±206.77	0.247
**TB(ummol/L)**	22.91±31.16	20.10±10.26	0.276
**SCr(ummol/L)**	70.23±20.72	60.48±18.86	<0.001
**Na(ummol/L)**	138.53±4.85	140.56±3.64	<0.001
**ALB(g/L)**	32.83±4.26	32.53±4.37	0.599
**PT(s)**	14.90±1.90	14.84±1.84	0.811
**INR**	1.30±0.16	1.29±0.16	0.671
**D-dimer**	4.52±6.83	4.01±8.60	0.671
**NH3(ummol/L)**	35.85±25.57	33.95±22.81	0.069
**WBC(*10^9/L)**	3.50±1.98	3.57±2.71	0.832
**PLT(*10^9/L)**	100.40±80.68	92.01±72.39	0.400
**PV(cm/s)**	26.93±10.75	25.49±7.76	0.300
**SPV(cm/s)**	28.11±7.54	31.53±11.40	0.063
**Size: 6/7/8/10(mm)**	29/22/63/1	58/37/203/1	0.082

Values are presented as means ± SD. TBIL= total bilirubin; CTP= Child–Turcotte–Pugh; HE: Hepatic Encepholapathy; PT= prothrombin time; PLT= platelets; WBC= white blood cells; Post-TIPS HE= Postoperative hepatic encephalopathy with TIPS; TB: Total Bilirubin; ALB: Albumin; AST: aspartate transaminase; ALT: alanine transaminase; INR: international normalized ratio; Scr: serum creatinine; PVP: Portal Vein Preassure; MELD: Model of End-Stage Liver Disease; PV: Portal velocity; SPV: Splenic vein velocity.
